# Underwater low-light enhancement network based on bright channel prior and attention mechanism

**DOI:** 10.1371/journal.pone.0281093

**Published:** 2023-02-02

**Authors:** Zhangjing Zheng, Xixia Huang, Le Wang

**Affiliations:** Institute of Logistics Science and Engineering, Shanghai Maritime University, Shanghai, People’s Republic of China; Guru Ghasidas Vishwavidyalaya: Guru Ghasidas University, INDIA

## Abstract

At present, there are some problems in underwater low light image, such as low contrast, blurred details, color distortion. In the process of low illumination image enhancement, there are often problems such as artifacts, loss of edge details and noise amplification in the enhanced image. In this paper, we propose an underwater low-light enhancement algorithm based on U-shaped generative adversarial network, combined with bright channel prior and attention mechanism, to address the problems. For the problems of uneven edges and loss of details that occurred in traditional enhanced images, we propose a two-channel fusion technique for the input channel. Aiming at the problems of brightness, texture and color distortion in enhanced images, we propose a feature extraction technique based on the attention mechanism. For the problems of noise in enhanced output images, we propose a multi-loss function to constrain the network. The method has a wide range of applications in underwater scenes with large depth. This method can be used for target detection or biological species identification in underwater low light environment. Through the enhancement experiment of underwater low light image, the proposed method effectively solves the problems of low contrast, blurred details, color distortion, etc. of underwater low light image. Finally, we performed extensive comparison experiments and completed ablation experiments on the proposed method. The experimental results show that the proposed method is optimal in human visual experience and underwater image quality evaluation index.

## 1. Introduction

Underwater images are an important carrier and presentation of underwater information. However, due to the influence of many factors such as insufficient irradiance and particles in water, underwater images are characterized by low contrast and unexpected noise. Underwater low-light images can not only cause unpleasant subjective visual experiences, but also affect the implementation of many vision-based technologies, such as underwater target detection and tracking. Therefore underwater image processing is important for ocean exploration and development, underwater rescue, etc. Underwater low-light enhancement, as a subfield of image enhancement, is widely used in the pre-processing of underwater target detection, etc. The enhanced underwater images will significantly improve the detection effect, convey the correct information, and enhance the application of computer vision in underwater.

The underwater image low-light enhancement algorithm is based on the research of low-light enhancement algorithm in land scene and color enhancement algorithm for underwater images. Using the imaging model of underwater images, we combine the low-light enhancement algorithm in land scene to study the underwater low-light image enhancement. Low-light image enhancement algorithms and underwater color correction studies mainly divided into two main categories: traditional-based enhancement methods and deep learning-based enhancement methods. Based on the traditional enhancement methods, the low light image is enhanced mainly by using the dehazing physical model, HE, and retinex. For example, Dong et al [1] proposed a method based on dark channel prior. [2–4] proposed a method based on histogram equalization, and [5–9] proposed a method based on retinex theory. The dehazing physical model algorithm enables researchers to stimulate new ideas on low illumination enhancement. However, due to the difference between underwater low-light scene and land scene in the imaging process, it is easy to misestimate the transmittance and atmospheric light value, which also limits its enhancement effect to some extent. Histogram equalization (HE) methods enhance the contrast of low-light images by stretching the dynamic range to make the gray distribution of the output image falls within the range of [0, 1], which avoids the problem of losing picture details due to the saturation of relatively bright areas when the low light image is directly enlarged, but such methods usually only adjust the contrast of the whole image globally, so the difference of local brightness is ignored. However, there are many impurities and particles in the underwater low-light scene, resulting in the abnormal local gray value in a wide range, so that the contrast of underwater low-light image cannot be properly restored, which may lead to insufficient or excessive brightness of the output image, or even generate strong noise. The enhancement algorithm [9] proposed based on Retinex theory is to decompose the image into illumination and reflection components. It performs joint light adjustment and noise suppression through adaptive adjustment to achieve the effect of low light enhancement. This kind of method is effective in adjusting light and removing small noises. However, the attenuation rate of the light intensity in the underwater low light scene is quite different from that in the land scene, and there is a large noise interference. At the same time, because such methods are only restricted by manual adjustment, the adaptive capability is not strong, intensive noise still exists in the enhancement results, and sometimes the illumination of local details is insufficient or excessive. The methods of these physical models have achieved certain results in the field of image enhancement in their respective periods, but they are often applied to a single scene, when applied to different enhancement targets, they need to be combined with specific research objects to make certain modifications to their theoretical models. In addition, the literature [10–12] also proposed image quality enhancement models including underwater image enhancement that mimic biovision. These models, inspired by biological vision, perform image quality enhancement by simulating the way of information transmission between atmospheric light and retinal cells or cells within the retina, which not only achieve very good results but also are very innovative. For example, Zhang et al. [12] proposed to roughly remove the low-frequency content of the haze with bipolar cells, modulate the output of the cone bipolar cells with long-free protrusive cells, and compensate the loss of details by increasing the image contrast. Then RGC was used to refine the local haze removal effect as well as the enhancement of image details, and finally, very good image quality results were achieved. However, the image enhancement effect of these methods in underwater low-light environment needs to be further enhanced.

**Fig 1:**
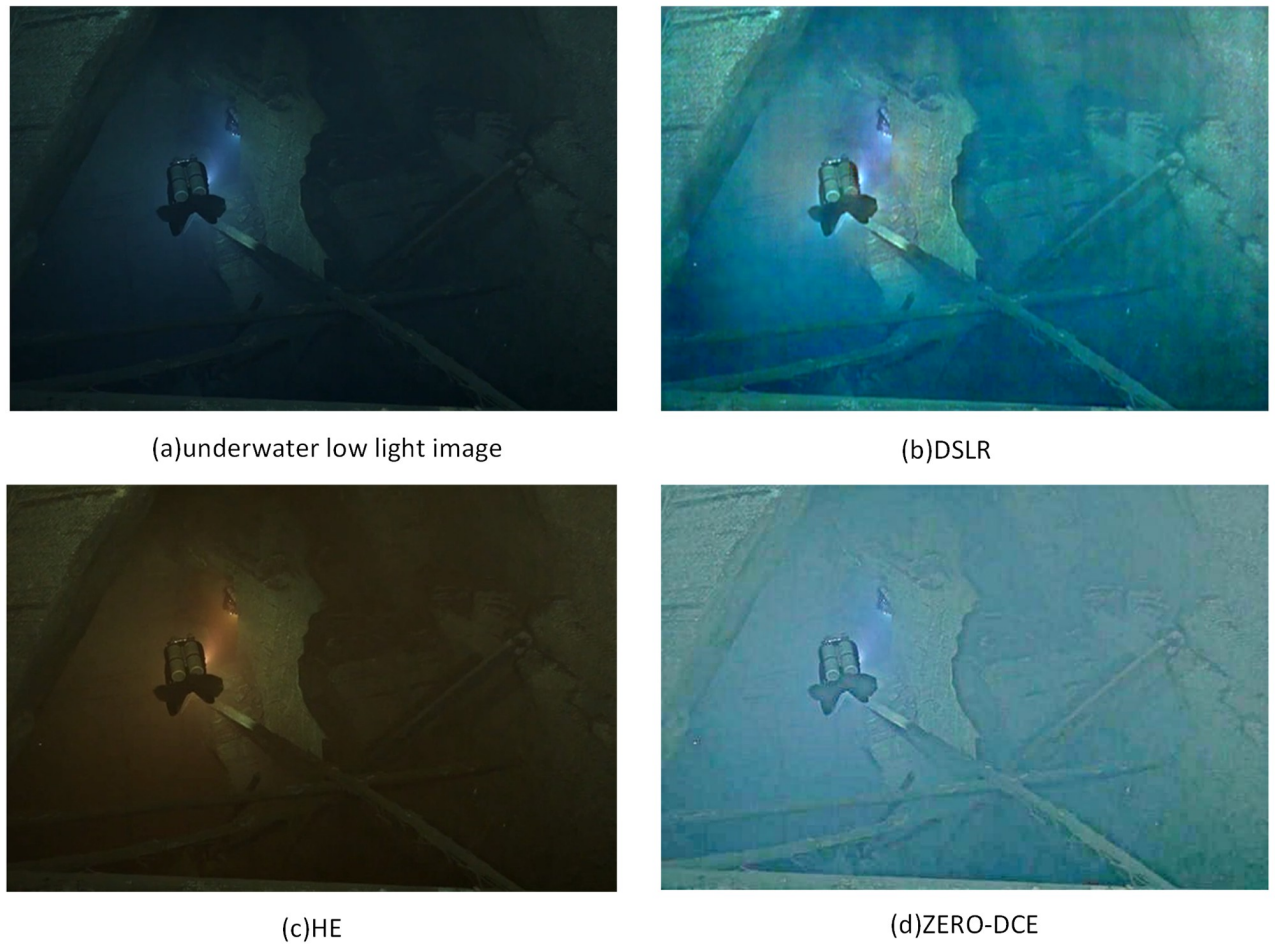
The enhancement effect of current low-light enhancement algorithms on underwater low-light images.

In deep learning-based approaches, the models are usually trained using convolutional blocks using a huge database. Deep learning-based enhancement algorithms are mainly based on two frameworks, namely, convolutional neural networks (CNN) and generative adversarial networks (GAN). For example, the literature [13–15] proposed a CNN-based approach and the literature [16–18] proposed a GAN-based approach. Among these methods, Literature [15, 17] based on well-prepared paired images and well-designed models can achieve recovery exquisite restoration of detail by relying on huge datasets. But the existing loss functions do not correspond well to human perception, and there are differences between the synthetic dataset and the real image, which lead to unsatisfactory visual effects, and the enhanced image may have color distribution offset and residual noise. At the same time, getting pairwise images in an underwater scene is an impossible task. The literature [18] does not require the construction of paired datasets, reducing the reliance on synthetic datasets. However, some of the fine details of the low-light images are not easily recovered and strong noise still appears in the enhancement results without paired supervision. Fig 1 illustrates the limitations of existing methods. For example, method [4] found that they cannot achieve correct image color restoration when processing underwater low light image enhancement. Although the method [19, 20] can enhance the contrast of underwater images and restore colors to a great extent, however, there are still color artifacts in the enhanced image, and the detail texture part is not very delicate.

Most of the above methods are designed for low light enhancement of images in land scenes and color correction of underwater images in shallow water. At present, no research exists on enhancement algorithms for low-light images underwater, when using low-light enhancement algorithms in terrestrial scenes or underwater color enhancement algorithms applied directly to low-light image enhancement in deep water scenes, problems such as enhancement overexposure, halo artifacts, and loss of edge details are likely to occur. In addition, it is difficult to obtain paired images in underwater low-light scenes. Based on these two factors, we propose an enhancement method of dual channel GAN combined with an attention mechanism. First we use the bright channel, and then more features are acquired through dual-channel fusion. Then the attention mechanism is used in the generator to reconstruct the extraction mechanism of features and multiple loss functions are used for constraint training. Specifically, our contributions are mainly as follows:

1. Bright channel prior: It is proposed to extract the bright channel image using the bright channel prior knowledge, then extract the features of the original image and the bright channel image separately, and finally fuse the features of the dual channels to enrich the feature information.
2. Hybrid attention mechanism: A generative adversarial network incorporating an attention module is established, the feature information of color, brightness, texture and other channels in underwater low-light images can be accurately extracted by adding attention mechanism in the feature extraction stage, only the required features of each channel are retained and irrelevant features are discarded, and the feature information is selected effectively.
3. Multiple loss function: The loss function is a combination of self-feature retention loss and color invariant loss. It enables the network to consider the details, color authenticity and texture information of the image at the same time, and solve the problem of distortion and color deviation of underwater low-light enhanced images.

This paper is structured as follows: The first part describes the importance of underwater low-light enhancement research and the low-light enhancement research in terrestrial scenes and underwater color correction research related to this research, describing the challenge of underwater low light image enhancement and proposing solutions. The second part presents related works, it mainly includes underwater imaging models, image enhancement methods based on traditional methods and image enhancement methods based on deep learning. The third section illustrates our contributions to underwater low-light image enhancement methods through the design of network models, mainly including bright channel prior, attention mechanism, and multiple loss functions. In the last part, extensive contrast and ablation experiments were conducted. Through qualitative and quantitative experiments, it is proved that the proposed network is superior to several other low-light image enhancement networks. Meanwhile, it is also proved that the designed network methods are effective for image enhancement in underwater low-light image scenes. The underwater low light image will be enhanced from two aspects of visual experience and image quality evaluation index.

## 2. Related work

In this section, the current low light enhancement algorithms and underwater color enhancement algorithms are summarized. The underwater imaging model will be introduced first, and then the image enhancement algorithm based on traditional methods and deep learning will be introduced.

### A. Underwater imaging model

**Fig 2:**
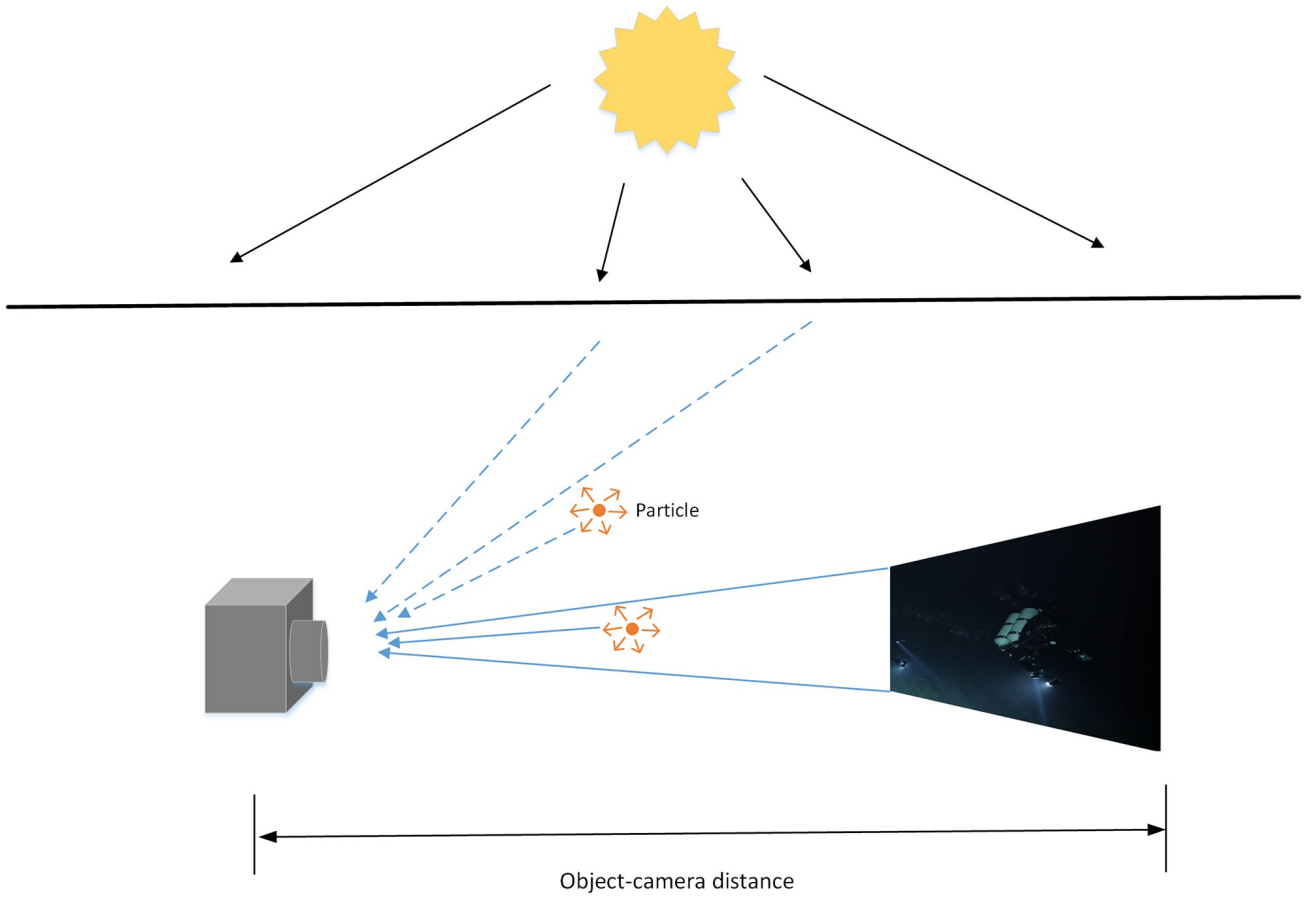
Underwater image imaging model.

Firstly, according to the Jaffe-McGlamery [21] underwater imaging model, the underwater image can be represented as a linear combination of three components, which are the direct component, the forward scattering component and the backward scattering component. Among them, the forward scattered light, which is reflected from the target surface or scattered by the suspended water, will lead to the fuzzy phenomenon of the image acquired by the imaging system. Backscattered light is the natural light incident on water and scattered by suspended particles into the imaging system. It will result in low contrast of the image obtained by the imaging system. The imaging schematic of the underwater image is shown in Fig 2.

Normally, the forward scattering component will be ignored due to the small distance between the camera and the target object, so the model of underwater imaging can be simplified as:

$$\text{I}\left(\text{x}\right)=\text{J}\left(\text{x}\right)\text{t}\left(\text{x}\right)+\text{B}\left(1-\text{t}\left(\text{x}\right)\right)$$ (1)

where I(x) denotes the original image, J(x) denotes the clear image, J(x)t(x) denotes the direct component, B(1-t(x)) denotes the backward scattering component, B denotes the underwater ambient light, and t(x) denotes the scene light transmittance. Since the absorption rates of water for different light are different and t(x) for different channels of light are different, the imaging model can be further expressed as:

$$I_{c}\left(x\right)=J_{c}\left(x\right)t_{c}\left(x\right)+B_{c}\left(1-t_{c}\left(x\right)\right)$$ (2)

where c ∈ {R,G,B} denotes the different color channels.

### B. Proposed method

#### 1. Traditional low-light image enhancement methods

For the traditional low-light image enhancement methods, they can be mainly divided into the dehazing-based physical model algorithm, the histogram equalization (HE), and the Retinex method. The algorithm based on dehaze physical model is an enhancement algorithm based on atmospheric scattering model. In 2011, Dong et al [1] found the similarity between low-illumination images after inversion and hazed images, and provided an indirect enhancement idea for low-illumination enhancement by referring to the dark channel a priori theory adopted by the dehazing image algorithm [22]. However, due to insufficient constraints, it is easy to have errors in the estimation of transmittance and atmospheric light value, and artifacts often occur in the enhancement results. To address these problems, Chiang et al [23] proposed a wavelength compensation and image dehazing algorithm (WCID) to eliminate enhancement distortions caused by light scattering and color variations. The method achieves this by removing the possible presence of artificial light sources. Peng et al [24] proposed to introduce a fuzzy prior to estimate the scene distance by image blurring, but did not fully consider the transmission spectral dependence; Berman et al [25] proposed that, based on the de-fogging model, by estimating the attenuation rates of two additional global parameters—blue-red and blue-green color channels, the restoration problem of underwater images was simplified to single image dehazing. These improved algorithms improve the problem of estimating atmospheric light values and transmittance to some extent, but still suffer from ill-defined edges, halo artifacts, and image overexposure.

Histogram equalization-based algorithms improve the image contrast by making the uneven histograms evenly distributed by mapping changes. To improve over-enhancement of local image area by HE algorithm, Pizer et al [2] proposed the enhancement algorithm of Adaptive Histogram Equalization (AHE), by calculating the local histogram of the image, the local contrast of the image is effectively improved by redistributing the brightness. But the processing speed of this algorithm is not good. To improve the performance algorithm, Zuiderveld [3] proposed Contrast Limited Adaptive Histogram Equalization (CLAHE), which uses linear interpolation to improve the processing speed of the AHE algorithm and overcome the limitations; Hitam et al [4] proposed a hybrid restricted contrast adaptive histogram algorithm, which uses Euclidean parametrics to fuse the enhancement results of HSV and RGB channels of underwater images based on CLAHE, to some extent, this method can suppress the noise and color distortion of the enhanced image. However, the improvement effect of HE is very limited for plots with complex depth of field and uneven blurring degree.

Retinex-based algorithms are used to enhance low-light images by removing the illumination component from the image to obtain the reflectance component characterizing the image eigenvalue. Retinex is based on color sense consistency (color constancy), which balances dynamic range compression, edge enhancement, and color constancy, therefore, various types of images can be enhanced adaptively. Jobson et al [5, 6] proposed the most popular single-scale (SSR) and multi-scale Retinex algorithms and specified the importance of log transform and Gaussian filtering for illumination estimation, but using reflectance as the final enhancement result often leads to over-enhancement. Fu et al [7] proposed a method to adjust illumination by fusing multiple derivatives of the initial estimated illumination map (MF), and the enhancement of MF effect is better. However, due to the blindness of the illumination structure, MF may lose the realism of texture-rich regions. In addition, Zhang et al [8] proposed an underwater image enhancement algorithm with extended multiscale Retinex for the halo problem, by extending the MSRCR algorithm to CIELAB color space, it can effectively suppress the halo phenomenon in the process of image enhancement. Retinex such algorithms can largely adjust the grayscale distribution of the image better, but cannot improve the image details well, and easy to produce color bias and color distortion phenomenon. On the enhancement and improvement algorithm of multi-scale Retinex algorithm with good processing effect, because of the complexity of the algorithm and its high time complexity, the real-time performance of the algorithm is not strong.

Among the various traditional low-light image enhancement methods, the physical models designed in most of the methods can better fit the image contrast and saturation changes brought by exposure values in the land scene, but the differences between land scenes and underwater scenes in the imaging process cause frequent image overexposure, color distortion, and poor detail processing when these methods are applied to underwater scenes, which makes it difficult for traditional image enhancement methods to have a better application in image enhancement of underwater low-light scenes.

#### 2. Low-light image enhancement method based on deep learning

The algorithms of traditional low-light image enhancement methods has strong interpretation, but when faced with different enhancement goals, the model is not robust enough, and it is difficult to achieve good enhancement effect. With the rapid development of deep learning, more and more low-light image enhancement methods based on deep learning are proposed.

Deep learning-based image enhancement algorithms are broadly classified into Convolutional Neural Networks (CNN)-based approaches and Generative Adversarial Networks (GAN)-based approaches, which train models from a large database and then process the test images in the input. Hou et al. [13] proposed an underwater image enhancement network based on joint residual learning, modeling the underwater image enhancement task as simultaneous learning of transmission map and scene residual, and the model includes a data-driven residual structure for transmission map estimation and a knowledge-driven scene residual computation method for underwater illumination balancing. Wang et al. [14] proposed an end-to-end underwater image enhancement network, noted as UIE-Net, which mainly consists of a color correction sub-network and a dehazing sub-network. The network can learn the effective feature representations of both color correction and dehazing tasks simultaneously, which improves the convergence speed and accuracy of the algorithm. However, due to the special underwater environment, it is impossible to obtain the real scene without water, so there is a lack of real reference images when evaluating the enhanced output of underwater low-light images. Lv et al [15] proposed the CNN-based MBLLEN method, and the core idea of this algorithm is to extract rich image features at different levels in different classes, do image enhancement by multiple sub-networks. Finally, the output image is generated by multi branch fusion, and the image quality is improved from different aspects. The algorithm can be used not only for image enhancement, but also for video enhancement. Based on generative adversarial networks, Liu et al [16] proposed a multi-scale feature fusion network based on conditional generative adversarial networks (cGAN) for underwater image color correction (MLFcGAN), This algorithm fuses the global features with the local features at each scale to achieve better performance in color correction and detail retention. Lu et al. [17] proposed a multi-scale CycleGAN network for underwater image restoration, which realized the combination of dark channel prior and CycleGAN, this algorithm is effective in contrast enhancement and color correction. Jiang et al. [18] proposed an unsupervised low light image enhancement method EnlightenGAN based on GAN, such algorithms require large data sets for training and testing. However, the current low-light image dataset is limited, and the low-light image synthesized by synthesis differs from the real low-light image.

Based on the above analysis, the current deep learning-based image enhancement methods applied to underwater low-light scenes have two main defects: first, it is difficult to collect a large number of paired data sets in underwater low-light scenes, which cannot be applied to CNN-based supervised learning networks; second, because underwater scenes are more complex than land scenes, the extraction of various feature information by GAN network-based methods is not accurate enough, this will result in the enhancement not being good enough.

## 3. Method

In this section, an image enhancement network for underwater low-light scenes is proposed, aiming at the enhancement of underwater low-light images. The overall network structure designed based on the generative adversarial network model, and the most critical proposed bright channel prior knowledge, hybrid attention mechanism, and multi-loss function proposed for underwater low-light scene enhancement tasks will be introduced in detail.

### 3.1 Network structure

**Fig 3:**
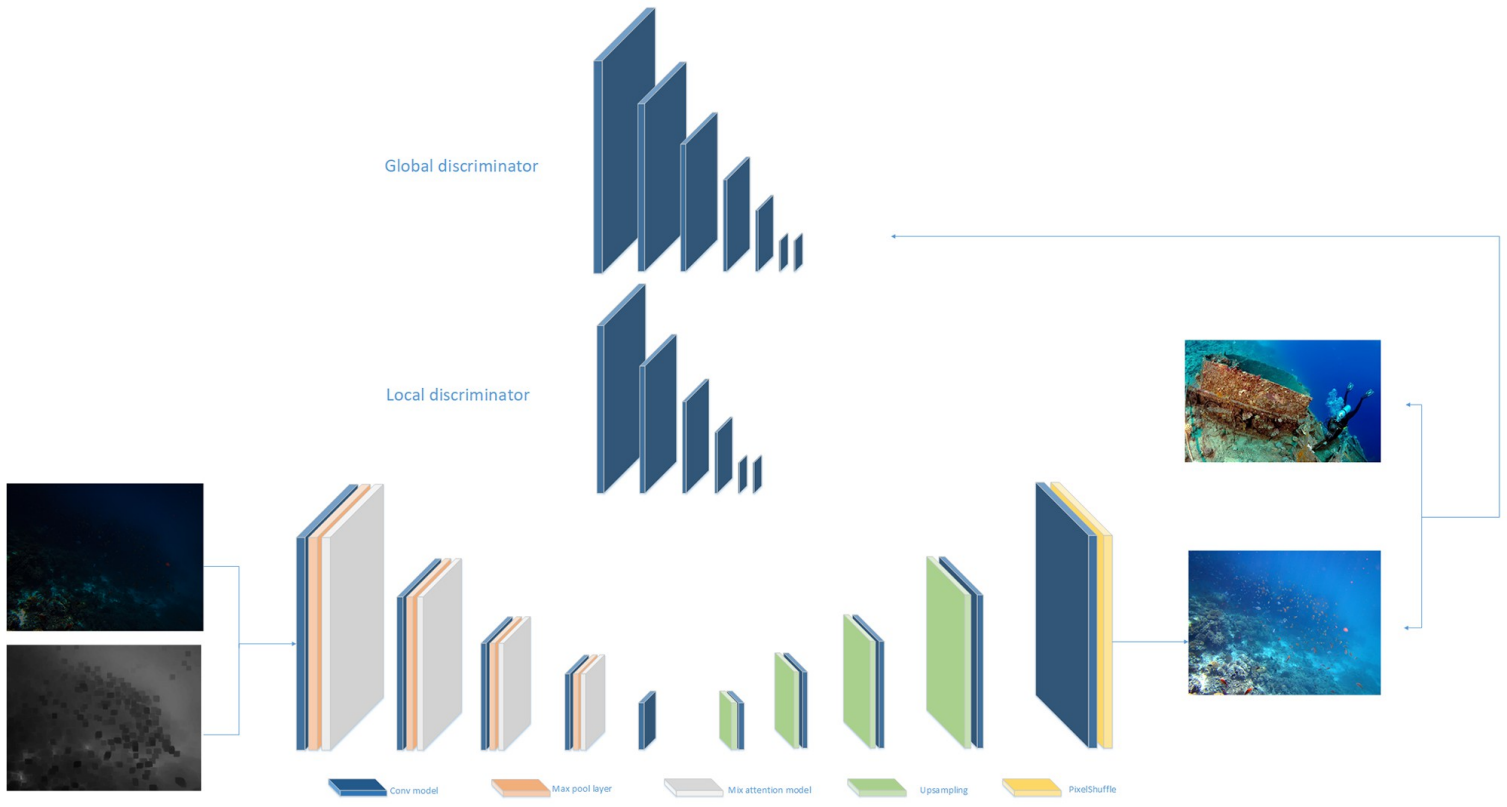
Network structure diagram.

As shown in Fig 3, considering the difficulty of obtaining paired datasets in underwater low-light scenes and in order to obtain better image enhancement. Our network structure takes GAN as the framework, and constructs the generator and discriminator modules. Among them, for the need of extracting many complex features such as brightness, chromaticity and saturation in underwater low-light images, we choose U-Net [26] as the generator, which can not only extract features at different levels, but also interact and fuse features at different levels by decoder and skip connection, the discriminant network uses a combination of global discriminator and local discriminator. In particular, for the complexity of the image enhancement task of underwater low-light scenes, the prior knowledge of the bright channel is used at the input the addition of a hybrid attention mechanism in the generator, and the use of multiple loss functions at the end of the network for optimization, all of which will be described in detail later.

### 3.2 Bright channel prior

In order to improve the image enhancement effect in underwater low-light environment, it is proposed that the underwater low light images should be subject to a prior processing of bright channels before feature stitching, in order to solve the problem of noise and loss of details of the output image caused by insufficient feature extraction in the current depth learning network. Underwater low-light images often show color distortion and low contrast because the imaging model of underwater low-light images is similar to the hazy model of land scenes, which is composed of direct signal and background scattering signal, and the usual way to solve such morbid problems is to insert priori information. Based on this, a prior method of bright channel is proposed for underwater low light image enhancement. The bright channel prior [27] is inspired by the dark channel prior. The dark channel priori believes that in most non sky local area images, the dark primary colors of these scenes are always gray, so there is at least a color channel with very small intensity values and tends to zero. Similarly, a priori knowledge of the bright channel suggests that for clear well-lit images, there is always at least a color channel with a large intensity value tending to 255 in each pixel region.

**Fig 4:**
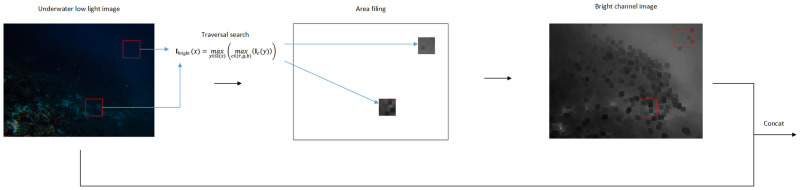
The filling process of the bright channel image.

Underwater low-light images, compared with clear normally illuminated images, have lower bright channel values. On the one hand, it will cause image noise to be amplified and the image details to be lost. On the other hand, it will cause insufficient feature extraction in the learning process of the network. To address these two points, It is proposed for the first time to embed the prior knowledge of bright channel into underwater low light image enhancement. First, obtain bright channel images by using bright channel a priori knowledge, then extracting features of the original image and bright channel images separately. Finally, the two types of features are fused to enrich feature information. The specific process of our proposed bright channel method is shown in Fig 4, which first finds the maximum brightness pixel points of the underwater low-light image by traversing the sub-region, uses the maximum brightness of the sub-region to build a bright channel image with the same structure as the original image. Then the extracted bright channel image features are spliced with the original image features and sends it to our designed generative adversarial network for training. The proposed bright channel prior scheme, it will effectively solve the problem of losing feature information such as chromaticity, contrast, saturation, and fine granularity in underwater low-light scenes due to insufficient luminance, and improve its image enhancement effect to a certain extent. Among them, the prior knowledge of bright channel for underwater low-light images can be defined as:

$$I_{\text{bright}\;}\left(x\right)=\underset{y\in \Omega \left(x\right)}{max}\left(\underset{c\in \left(r,g,b\right)}{max}\left(I_{c}\left(y\right)\right)\right)$$ (3)

Where **I**_bright_ (*x*) represents the bright channel image and **I**_*c*_ (*y*) is the underwater low-light image. Eq (1) ensures that the luminance value of each pixel region of the bright channel image is not smaller than the input image, and the bright channel image has a certain spatial smoothness and is similar to the original image structure.

### 3.3 Hybrid attention module

Aiming at the problem of color difference and noise of low light enhancement output image, a hybrid attention module is proposed, which can restrain the color deviation and noise of output image. In low-light images, a larger receptive domain is the key to reduce color artifacts [28, 29], because broader information can guide the network to learn what it should learn in the face of severe noise. In the traditional method, the method of expanding the receptive domain is simply overlaying the residual blocks. In the feature extraction of the traditional network, there is no differentiated operation of feature extraction due to the different contribution of each channel to the subsequent network, and the input harmful features and favorable color information are extracted together by indistinguishable feature extraction. Therefore, when enhancing the color information of the image, the noise information contained in the image may also be amplified. The channel attention module carries out differentiated feature extraction operation by modeling the interdependence between individual channels to enhance the color reality and noise suppression of the output image.

**Fig 5:**
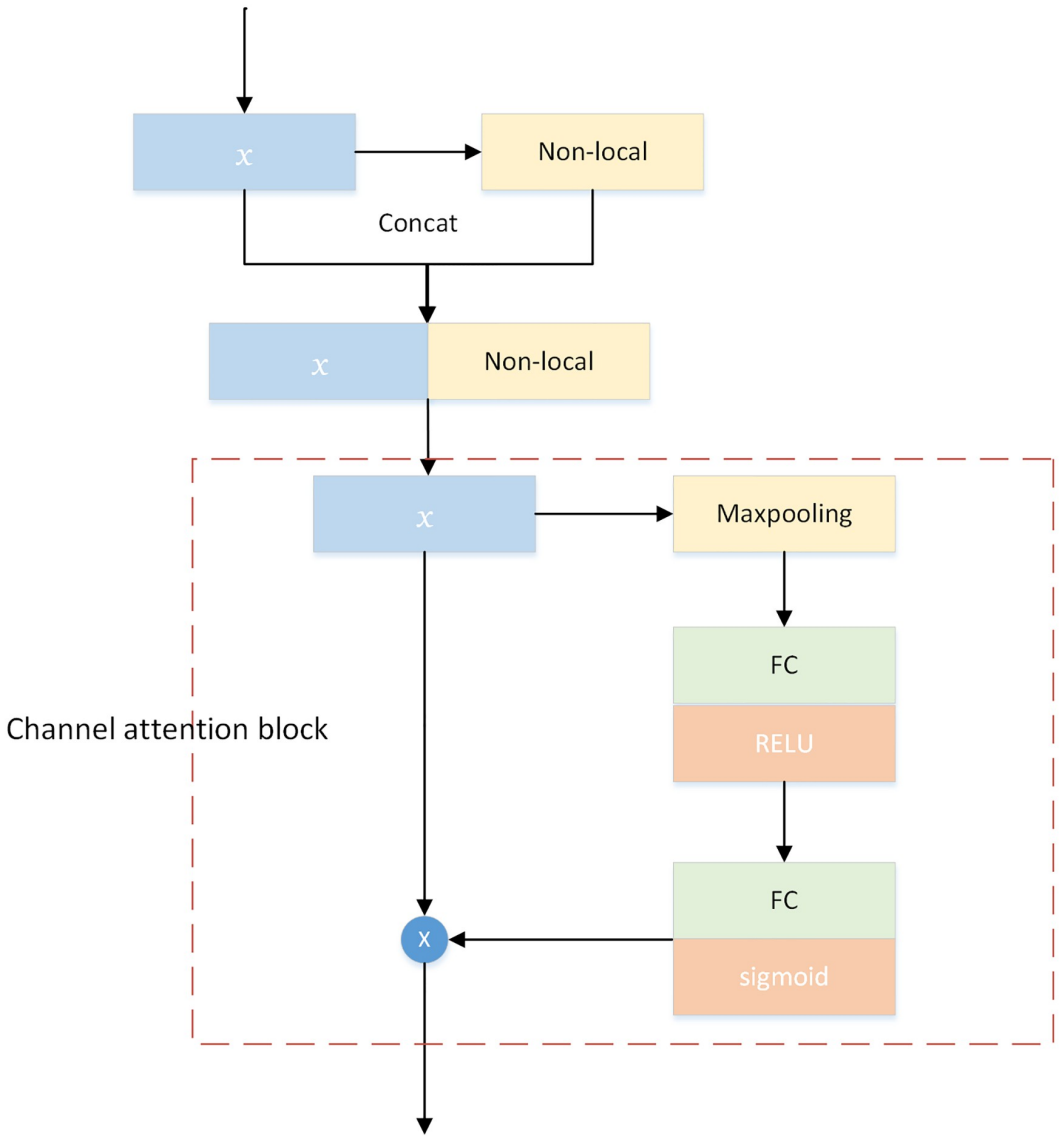
Flow chart of feature extraction mechanism after adding hybrid attention module.

The main purpose of adding the hybrid attention module to the generative adversarial network is to model the dependencies among the channels. The flowchart of the feature extraction mechanism of the hybrid attention module is shown in Fig 5. The feature maps are extracted in the LR space [30]. Firstly, a nonlocal operation [31] is taken to obtain features with a larger range of information in the spatial domain, non-local operations are designed to enhance the feature representation capability of the network. Non-local operations are used to aggregate the information of different positions in the feature map so that the network has a global acceptance domain. The spatial attention module mainly uses the non-local correlation of the image to remove noise. We connect the acquired features and feed the connected features to the channel attention block to generate the final feature representation. In the channel attention block, the maximum pooling layer is first used to obtain the representative values in each channel, and then two fully connected layers and activation functions are used to understand the meaning between channels. The channel attention module has good motivation, which not only eliminates the harmful features of the input, but also highlights the favorable color information to guide the network to refine the redundant color features. The channel attention module as well as the spatial attention module can take into account both local and global information and together achieve the suppression of chromatic aberration and noise. Through the hybrid attention module, the network can make full use of the information of different channels and positions in the feature map to accurately extract the required feature information.

### 3.4 Loss function

For the problem of distortion and color bias in underwater low-light-enhanced images, the use of self-feature preservation loss [18] and color constancy loss [19] is proposed for joint optimization, which can enable the designed network model to consider the image details, color fidelity, and texture information simultaneously, and the joint loss function will be described in detail in this section.

#### 3.4.1 Self-feature retention loss

For unsupervised networks trained with unpaired datasets, due to the lack of strict supervision information, the enhanced images often appear detail distortion, artifacts and other phenomena. To solve this problem, the self-feature preserving loss function [18] inspired by the VGG [32] model can preserve the image features before and after enhancement to itself. Similarly, setting the self-feature retention loss of the local discriminator can capture the perceived difference between the output image and the real image, and improve the discriminator’s ability to detect forgeries and thus optimize the generator more accurately.

Self-feature retention loss:

$$L_{SFP}\left(I^{L}\right)=\frac{1}{W_{i,j}H_{i,j}}\sum_{x=1}^{W_{i,j}}\sum_{y=1}^{H_{i,j}}{\left(\phi_{i,j}\left(I^{L}\right)-\phi_{i,j}\left(G\left(I^{L}\right)\right)\right)}^{2}$$ (4)

where *I*^*L*^ denotes the input low-light image, *G*(*I*^*L*^) denotes the output after generator enhancement, and *ϕ*_*i*,*j*_ denotes the extracted feature map. *i* denotes the i-th maximum pooling layer, and *j* denotes the j-th convolutional layer after the i-th maximum pooling layer. *W*_*i*,*j*_ and *H*_*i*,*j*_ are the dimensions of the extracted feature map.

#### 3.4.2 Loss of color constancy

Color bias is a long-standing problem in image enhancement tasks. In the underwater low light scene, the color deviation of the enhanced image is more prominent due to the extremely low light environment and the complex background scattering signal. Based on this, color constancy loss is proposed to solve the color deviation problem in the underwater low-light scene image enhancement task. It will try its best to maintain the color authenticity of the enhanced image. The color constancy formula can be expressed as follows:

$$L_{col}=\sum_{\forall \left(p,q\right)\in \varepsilon }{\left(J^{p}-J^{q}\right)}^{2},\varepsilon =\left\{\left(R,G\right),\left(R,B\right),\left(G,B\right)\right\}$$ (5)

where *J*^*p*^ is the average intensity value of channel p in the enhanced image, and (p, q) is a pair of channels.

#### 3.4.3 Joint loss function

Among the above loss functions, the contribution values of different loss functions to the learning ability of the network may be inconsistent. In order to solve this problem, we use the joint loss function to train the whole network. The distortion and color deviation of the enhanced image can be considered at the same time to optimize the color reality and texture details of the output image and the joint loss function is expressed as follows:

The self-feature retention loss function adopted by the whole network:

$$L_{SFP}=L_{SFP}^{Global}+L_{SFP}^{Local}+L_{G}^{Global}+L_{G}^{Local}$$ (6)

where $L_{G}^{Global}$ is the least squares loss function of the global discriminator, $L_{G}^{Local}$ is the minimum loss function of the local discriminator, $L_{SFP}^{Global}$ is the self-feature preserving loss function of the global discriminator, and $L_{SFP}^{Local}$ is the self-feature preserving loss function of the local discriminator.

The joint loss function adopted by the whole network:

$$L_{\text{total}}=W_{\alpha }L_{col}+W_{\beta }L_{SFP}$$ (7)

where *W*_*α*_, *W*_*β*_ denote the hyperparameters of the importance of the $L_{SFP}$ and *L*_*col*_ loss functions to the overall loss contribution, respectively, which can be adjusted, during training.

## 4. Experiments

### 4.1 Dataset

Our dataset consists of two parts, normal light images and low light images, considering the difficulty in acquiring paired underwater normal light images and low light images, an unsupervised image enhancement network is designed. In the whole network training process, paired normal light and low light image datasets are not required. The low-light images consist of two parts: underwater images taken under ultra-low light environment and some land dark light images selected from the SID dataset [33], while the normal light images are used to obtain normal light images taken under land scenes. Overall, our dataset contains a total of 836 low-light images and 1016 normal-light images.

### 4.2 Comparative experiment

**Fig 6:**
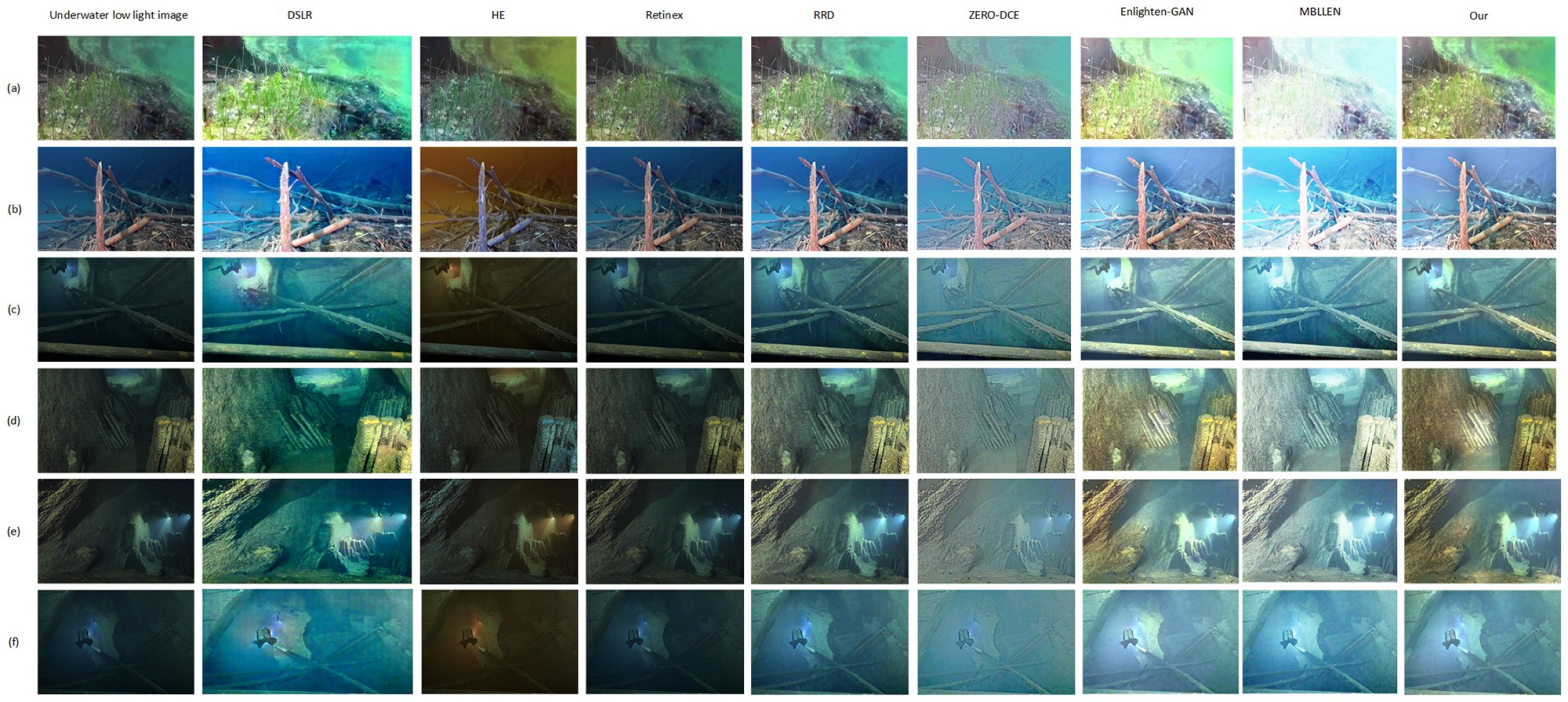
Comparison experiment between the proposed method and the methods in this paper.

To demonstrate the effectiveness of our proposed network, the designed network was compared with the seven most widely available networks on the same training and test sets, including Retinex [34], HE, and DSLR [20] based on physical models, and MBLLEN [15], Enlighten-GAN [18], RRDNet [35], and ZERO-DEC [19] based on deep learning models. In the experimental results, all images before and after enhancement are compared. The effect of image enhancement is evaluated by using the common no-reference image quality evaluation indicators NIQE and the underwater image quality evaluation indicators UICM and UIQM. and the final experimental results showed that the method proposed in this paper outperformed both the human eye visual experience and the image quality evaluation indexes of other networks. Carry out all our experiments on the Python 1.9.1 deep learning network framework. The network is trained on a PC with Nvidia GeForce RTX 2060 GPU, each group of experiments trained same number of times, all for 200 epochs, and then select the same test set for testing. In the training of our network, the batch size is 6, the initial learning rate is 0.0001, the Adam optimizer is used, whose beta1 parameter is taken as 0.5 and beta2 parameter is taken as 0.999, and the input images are uniformly scaled to 256*256 and initialized by kaiming. The test results of the network after training with each comparison network are shown in Fig 6.

Analysis of the enhancement effects of our network and the other seven networks in Fig 4 shows that our designed network outperforms several other networks in terms of brightness, contrast, and saturation of images. In general, the methods based on deep learning models outperform the traditional physical model methods in terms of visual experience. Specifically, the Retinex method, which is based on retinex theory, and its inability to apply to the harsh environment of low light underwater, thus showing insufficient brightness enhancement; DSLR suffers from local overexposure, insufficient local brightness, the entire image lacks smoothness. HE method not only has insufficient brightness enhancement, but also shows serious color deviation; Although RRDNet and ZERO-DCE are better than the physical model method to some extent, RRDNet lacks enhancement of naturalness and contrast, and ZERO-DCE lacks consideration of noise thus more noise appears in the enhancement process, which damages the fine-grained image. The Enlighten-GAN method is also achieving a good effect in enhancing underwater low light images, but the method presents a characteristic of slightly higher brightness and slightly lower contrast as a whole. The MBLLEN method is excellent in processing low light enhancement in land scenes, but when it is used to process low light images in deep water under complex environment, local exposure of the image is very serious, and the whole image presents a gray tone. In contrast, our network mainly guides the brightness enhancement of low-light images through the prior knowledge of bright channel. At the same time, the loss of color constancy is used to avoid the loss of color features in the enhancement process. In addition, the U-NET generator with hybrid attention mechanism is used to extract as much feature information as possible, so as to maximize the fine-grained, boundary information in the image enhancement process.

In addition, in order to evaluate the enhancement effect of various methods more scientifically, we calculate NIQE, UICM and UIQM image quality evaluation indexes before and after enhancement by various methods, as shown in Table 1. The data of various indexes in Table 1 conform to their distribution rules. For NIQE index, as a common index for quality evaluation of no-reference images, it can better reflect the quality of the enhancement effect. The smaller the index, the better the image enhancement effect. In comparative experiments, our average NIQE index reached 14.73, which is the best among all methods. UICM and UIQM are commonly used to evaluate the quality of underwater image enhancement. UICM represents the color fidelity of the enhanced image, while UIQM considers both the color fidelity, contrast and clarity. In the comparative experiment, our UICM and UIQM reached 13.13 and 2.27 respectively, both of which are the best. This is because the use of dual-channel rich feature number, attention mechanism to achieve feature differentiation extraction, the multi-loss function is used to optimize the network from various aspects such as color, texture and detail features, so as to enhance the color truth, contrast and clarity of the image to the maximum extent. In addition, various methods are compared in terms of running time and parameters, as shown in Table 1. Although the test time of a single image is not optimal, it also belongs to the short test time of various methods. Based on the above experimental results, it can be seen that the network effect proposed by us is the best in both subjective visual experience and image quality evaluation indexes.

**Table 1:** Image quality evaluation index of various methods on the same test set.

Method	NIQE	UICM	UIQM	Test time(s)
DSLR	22.46	6.07	1.01	0.46
HE	25.6	12.5	-0.53	0.173
Retinex	16.00	5.695	0.58	2.6
RRD	16.99	7.85	2.04	383
ZERO-DCE	16.91	7.46	1.59	0.003
Our	14.73	13.13	2.27	2.09

### 4.3 Ablation experiment

**Fig 7:**
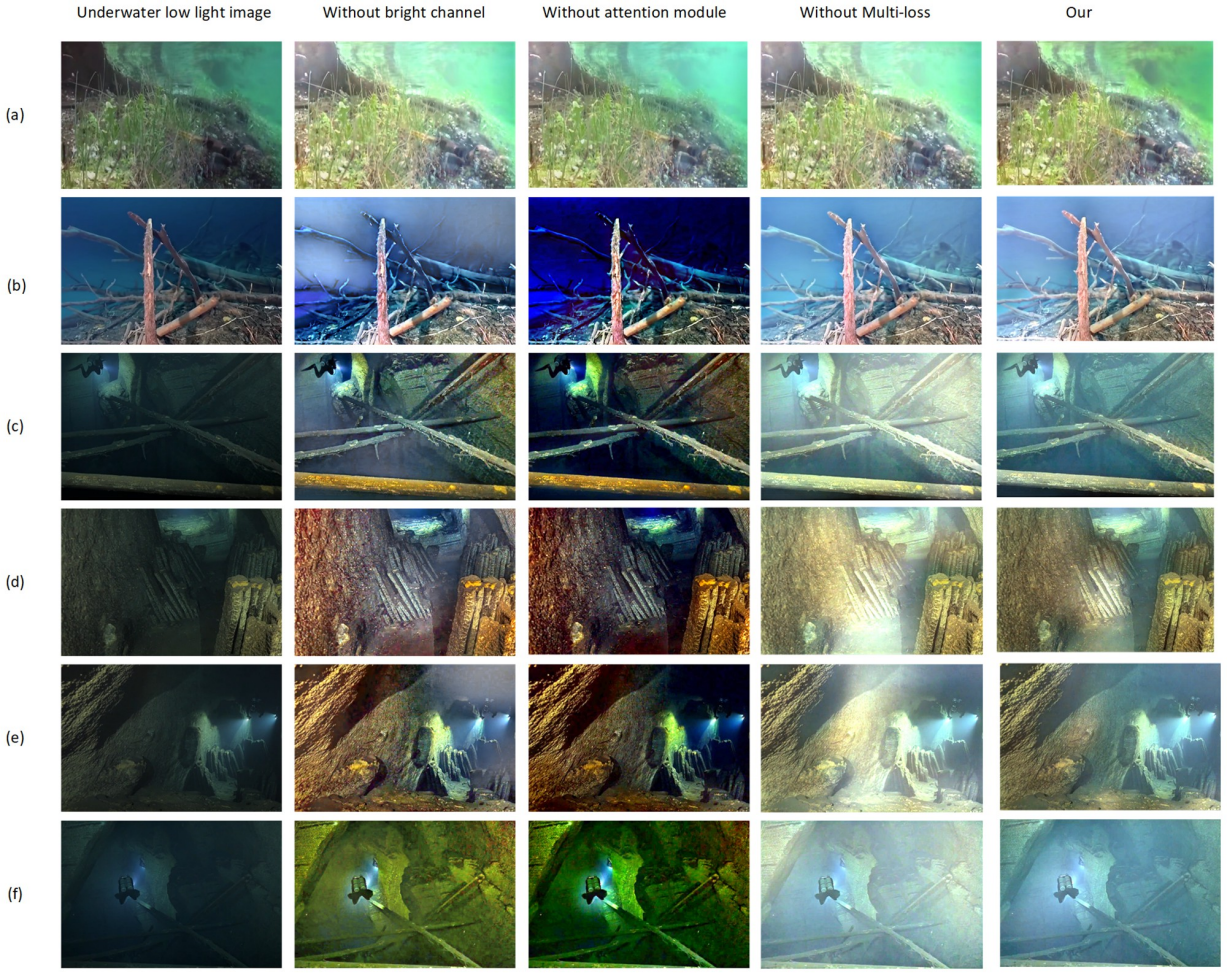
Ablation experiments, the second column shows that the bright channel prior method is removed from the overall network; the third column shows that the hybrid attention module is removed after removing the prior method of bright channel; the fourth column indicates that on the overall network, only the feature retention loss is used.

In this section, in order to verify the effectiveness of each part of our proposed methods for underwater low-light image enhancement, ablation experiments on bright channel priors, hybrid attention mechanism and joint loss function were conducted on the basis of GAN, respectively. During each ablation experiment, the hardware equipment was kept constant and the number of epochs trained was 200. After the training was completed, the same test images from the comparison experiments were used, and the test results are shown in Fig 7.

Fig 7 shows the influence of each part of the proposed method on enhancement effect on the same test set. From the experimental results, it can be seen that when the bright channel prior is absent, due to insufficient number of feature extraction it shows detail loss and background distortion; when the hybrid attention module is absent, the network is unable to suppress the harmful features, which results in color deviation and noise problems and serious background distortion; when only feature retention loss is used, each figure not only shows color deviation, but also carries certain detail distortion and noise interference. Therefore, the image in column 5 of Fig 7 has the best enhancement from a visual perspective. After removing each effective part of the method, each image shows different degrees of detail loss and color deviation, etc. The above ablation experimental results prove that all parts proposed in this paper are effective. Similar to the comparison experiments, we also tested the NIQE, UICM, and UIQM image quality evaluation indexes for each image in the ablation experiments, as shown in Table 2.

**Table 2:** Corresponding indexes of each method in the ablation experiment.

Method	NIQE	UICM	UIQM
Without bright channel	37.07	9.50	0.19
Without attention module	34.83	8.67	1.35
Without Multi-loss	40.03	6.61	-0.42
Our	14.73	13.13	2.27

According to the results of each index in Table 2, when the bright channel prior module is lacking, the NIQE index increases by 22.34, the UICM index decreases by 3.63, and the UIQM index decreases by 2.08, which means that the bright channel prior method is effective for the enhancement of underwater low-light images. On the basis of removing the prior of bright channel, when the hybrid attention mechanism module is lacking, its NIQE index increases by 20.1, the UICM index decreases by 4.46, and the UIQM index decreases by 0.92. It shows that the hybrid attention module used in this paper is also effective. When only the feature preservation loss function is used for optimization, its NIQE index increases by 25.3, and the UICM index decreases by 6.52, and the UIQM index decreased by 2.69. The above ablation experiment results prove that all parts proposed in this paper are effective from the perspective of image quality evaluation.

## 5. Conclusion

**Fig 8:**
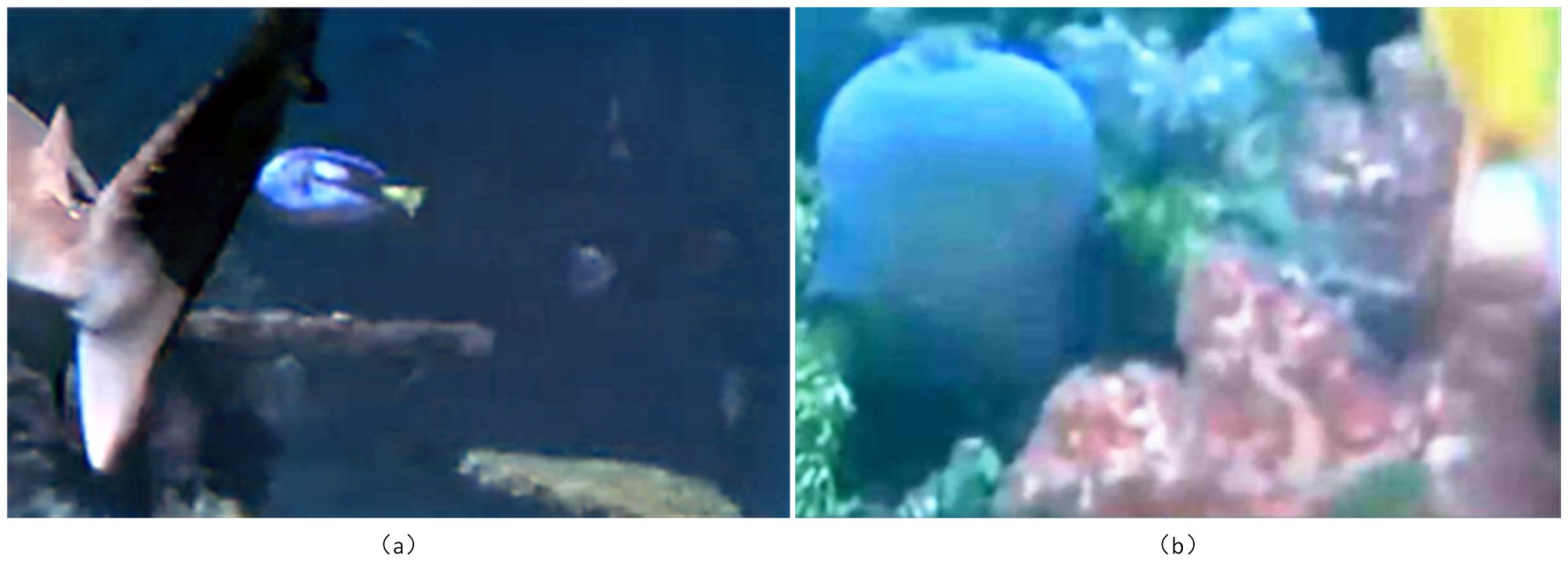
Imbalance between brightness enhancement and deblurring in a few images.

In this paper, aiming at the problems of enhancement overexposure, halo artifacts, edge detail loss, color distortion and so on which are easy to occur due to harsh environment in the enhancement process, an enhancement method of dual-channel U-type generative adversarial network combined with attention mechanism is proposed. In this method, the proposed bright channel prior solves the problem of feature extraction caused by insufficient brightness, the fused attention mechanism enables the network to extract the feature information of each part, and the joint loss function better solves the problems of color and detail distortion and noise interference that occur during enhancement. In the proposed method, the advantage is that we consider the enhancement task of the whole network from both brightness enhancement and image deblurring simultaneously, in addition to minimizing the distortion in the enhancement process as much as possible. The results of both comparison and ablation experiments demonstrate that our method is effective when applied to image enhancement in underwater low-light scenes, outperforming other methods currently available for low-light enhancement in underwater low-light scenes. However, our method still has shortcomings, a small number of images cannot balance the two tasks of brightness enhancement and deblurring. Refer to Fig 8 for details. When underwater low light images with many colors are involved, this method sometimes has poor enhancement effect in terms of clarity or brightness. The next work will focus on the research of the best balance between brightness enhancement and deblurring, so as to further improve the enhancement effect of underwater low light images.
